# Transcriptional profiling in *C. elegans *suggests DNA damage dependent apoptosis as an ancient function of the p53 family

**DOI:** 10.1186/1471-2164-9-334

**Published:** 2008-07-15

**Authors:** Sebastian Greiss, Björn Schumacher, Kaj Grandien, Jonathan Rothblatt, Anton Gartner

**Affiliations:** 1 Wellcome Trust Centre for Gene Regulation and Expression, School of Life Sciences, The University of Dundee, Dow Street, Dundee DD1 5EH, UK; 2 MGC Department of Cell Biology and Genetics, Center for Biomedical Genetics, Erasmus Medical Center, PO Box 1738, 3000 DR Rotterdam, The Netherlands; 3 Sanofi-Aventis Deutschland GmbH, Scientific and Medical Affairs, Biological Sciences Department, Industriepark Hoechst, Bldg. G879, Room 020, D-65926 Frankfurt am Main, Germany; 4 Sanofi-Aventis U.S., Scientific and Medical Affairs, Biological Sciences Department, 270 Albany St, Cambridge, MA, 02139, USA

## Abstract

**Background:**

In contrast to the three mammalian p53 family members, p53, which is generally involved in DNA damage responses, and p63 and p73 which are primarily needed for developmental regulation, *cep-1 *encodes for the single *C*. *elegans *p53-like gene. *cep-1 *acts as a transcription activator in a primordial p53 pathway that involves CEP-1 activation and the CEP-1 dependent transcriptional induction of the worm BH3 only domain encoding genes *egl-1 *and *ced-13 *to induce germ cell apoptosis. EGL-1 and CED-13 proteins inactivate Bcl-2 like CED-9 to trigger CED-4 and CED-3 caspase dependent germ cell apoptosis. To address the function of p53 in global transcriptional regulation we investigate genome-wide transcriptional responses upon DNA damage and *cep-1 *deficiency.

**Results:**

Examining *C. elegans *expression profiles using whole genome Affymetrix GeneChip arrays, we found that 83 genes were induced more than two fold upon ionizing radiation (IR). None of these genes, with exception of an ATP ribosylase homolog, encode for known DNA repair genes. Using two independent *cep-1 *loss of function alleles we did not find genes regulated by *cep-1 *in the absence of IR. Among the IR-induced genes only three are dependent on *cep-1*, namely *egl-1*, *ced-13 *and a novel *C. elegans *specific gene. The majority of IR-induced genes appear to be involved in general stress responses, and qRT-PCR experiments indicate that they are mainly expressed in somatic tissues. Interestingly, we reveal an extensive overlap of gene expression changes occurring in response to DNA damage and in response to bacterial infection. Furthermore, many genes induced by IR are also transcriptionally regulated in longevity mutants suggesting that DNA damage and aging induce an overlapping stress response.

**Conclusion:**

We performed genome-wide gene expression analyses which indicate that only a surprisingly small number of genes are regulated by CEP-1 and that DNA damage induced apoptosis via the transcriptional induction of BH3 domain proteins is likely to be an ancient DNA damage response function of the p53 family. Interestingly, although the apoptotic response to DNA damage is regulated through the transcriptional activity of CEP-1, other DNA damage responses do not appear to be regulated on the transcriptional level and do not require the p53 like gene *cep-1*.

## Background

Previous studies established the *C. elegans *system as a simple multicellular organism to study DNA damage responses and DNA damage induced apoptosis. These studies led to the identification of a core *C. elegans *DNA damage response pathway that includes conserved upstream DNA damage sensor molecules such as the PI3 kinase-like ATM and ATR (Ce-ATL-1) kinases, the Rad-17 clamp loader-like molecule and the Rad-9-Hus-1-Rad-1(Ce-MRT-2) (9-1-1) replication factor C like complex, all needed for DNA damage induced apoptosis, cell cycle arrest and DNA repair. In addition, a conserved checkpoint gene, *clk-2*, first discovered in *C. elegans *seems to act in a genetic pathway parallel to the 9-1-1 complex [1-4]. Besides these upstream signalling factors *C. elegans *also contains a single p53 family member termed *cep-1 *(*C. elegans *p53-like). This gene is involved in DNA damage induced germ cell apoptosis upon UV and ionizing irradiation (IR) and is required for mitotic germ cell cycle arrest upon UV treatment but does not overtly affect DNA repair as *cep-1 *mutant worms are only very weakly sensitive to UV and IR in organismal radiation survival assays [5-7].

CEP-1 sequence homology to p53 family members in other organisms is mostly restricted to the p53 DNA binding domain. CEP-1 acts as a transcription factor [7] and is able to bind to human p53 consensus sites and key features of DNA binding are conserved as shown by structural analysis [8]. It is unclear whether CEP-1 is functionally more related to mammalian p53 or to either of the two other mammalian p53 family members p63 and p73 that are implicated in DNA damage responses but also fulfil multiple developmental roles centred on epithelial development and neurogenesis respectively [9]. Although CEP-1 shows more sequence similarity to the p63 DNA binding domain [10] and p63 was recently shown to affect DNA damage induced germ cell apoptosis in mice [10], *cep-1 *was initially related to p53 based on its role in DNA damage induced apoptosis and the absence of an overt developmental phenotype. The assumption that *cep-1 *is more closely related to p53 than to the other p53 family members is further supported by the finding that both *C. elegans *BH3 domain-only proteins needed for efficient DNA damage induced apoptosis, EGL-1 and CED-13, are transcriptionally induced by *cep-1 *upon IR and UV treatment [5,11]. This response appears to be functionally equivalent to the p53 dependent transcriptional induction of mammalian BH3 only domain proteins like PUMA and NOXA [12,13] suggesting that the transcriptional induction of BH3 domain-only proteins by CEP-1 and p53 might be the central and conserved regulatory node to effect DNA damage induced apoptosis.

Here we take advantage of the *C. elegans *system which only contains one p53-like gene to address by transcriptional profiling, if *cep-1 *has additional functions in the DNA damage response apart from the transcriptional induction of *egl-1 *and *ced-13*. We did not find any genes transcriptionally regulated by *cep-1 *in the absence of ionizing irradiation (IR). Among the IR-induced genes, only three were dependent on *cep-1*. Two of those genes encode for BH3 domain proteins *egl-1 *and *ced-13*, previously implicated in worm germ cell apoptosis. These results, together with recent structural studies on CEP-1 [14] provide evidence that regulating DNA damage induced apoptosis might be an ancient function of the p53 family of transcription factors. *C. elegans *DNA repair genes are not transcriptionally regulated by IR, and IR-induced genes appear to be involved in general stress responses. Interestingly, there is an overlap between IR-induced genes and genes involved in aging.

## Results

To identify *cep-1 *dependent and IR dependent genes we chose to analyze the global gene expression profiles of developmentally synchronized *cep-1(lg12501) *worms approximately 24 hours post the L4 larval stage. At this stage somatic tissues of adult worms are postmitotic and at the same time the germ line is proliferative and already fully developed. Furthermore, at this stage embryos are developing in the uterus. For each experiment we independently grew wild type and mutant worms in triplicate and subjected them to IR by X-ray treatment (120 Gy) or to mock treatment and harvested worms for mRNA preparation 2 hours after IR. In previous studies we have shown that DNA damage induced apoptosis starts to occur two hours after IR and more importantly that transcription of *egl-1 *and *ced-13 *BH3 domain encoding genes is already activated at this time point in a dose dependent manner [11,15]. For our analysis we only considered genes whose hybridization signal on the *C. elegans *whole genome Affymetrix microarray was significantly above background levels (p < 0.05) in at least two out of three experiments for the condition under examination. Based on these criteria we could detect significant transcript levels of ca. 50% of all genes in untreated wild type worms (See Additional file 1).

We first examined IR-induced genes and found that 40 are induced more than 3 fold (p < 0.05) (Table 1) whereas 83 are induced more than 2 fold (p < 0.05) and 184 are induced more than 1.5 fold (p < 0.05) (See Additional file 2). We focused the analysis on the 83 genes that are expressed more than 2 fold upon X-ray treatment and confirmed these results by qRT-PCR with representative genes (Table 1). There are only 19 genes induced more than 5 fold and the strongest transcriptionally induced gene, upregulated approximately 100 fold, encodes a nematode specific protein of unknown function. We note that we obtained almost identical results when we treated worms with gamma irradiation (Cs 137 source), with 60 out of the 83 genes being induced by both X-rays and gamma-irradiation (Table 1, See Additional file 1). 23 of the 60 genes induced two hours after gamma-irradiation were still induced after 6 hours as determined by Affymetrix gene expression arrays with gamma-irradiated worms collected 6 hours after IR (Table 1, See Additional file 1). To our surprise the expression of only two of the IR-induced genes, namely *egl-1 *and *ced-13 *(a gene expressed at very low levels and only detected on arrays with gamma-ray treated worms) depends more than 2 fold on *cep-1 *(see below). The only gene found to be induced more than 2 fold by IR and thought to be linked to DNA repair was *pme-5*. This gene encodes for a tankyrase, a conserved protein characterized by both ankyrin repeats and poly-ADP-ribose polymerase motifs, which has previously been shown to be induced by IR in worms [16]. While human tankyrase has been implicated in telomere maintenance, its function in *C. elegans *is not known. Interestingly, *pme-5 *transcriptional induction by IR was reported to be dependent on the 9-1-1 DNA damage checkpoint complex component *hus-1*, which genetically acts upstream of *cep-1 *[16]. In the expression profiles we found that *pme-5 *induction was transient. It was upregulated two hours after IR but not 6 hours after IR. Upregulation was not dependent on *cep-1 *and *pme-5 *expression did not depend on the 9-1-1 DNA damage complex component *mrt-2 *(Table 2). We next asked whether further DNA damage response genes might be enriched upon IR by considering all 184 genes that are significantly induced by IR at least 1.5 fold but did not find any DNA repair genes (See Additional file 2). In summary, our results indicate that DNA repair genes are not transcriptionally regulated upon IR treatment in *C. elegans*. In contrast, in *Drosophila *there are several genes involved in DNA repair that are p53 dependent. Namely *RnrL*, the large subunit of ribonucleotide reductase, the DNA end joining enzymes ku70 and ku80, the recombination enzyme mre11, the mus205 polymerase zeta, and mus210 [17,18].

**Table 1:** Genes induced at least 3 fold 2 h after X-ray treatment

**name**	**X-ray 2 h**	**qRT-PCR**	**gamma-ray 2 h**	**gamma-ray 6 h**	**annotation**
**F49F1.6**	**155.32**	**134.9 ± 43.7**	**127.38**	**70.86**	**ShK domain-like, Secreted surface protein**
**K08D8.5**	**10.03**		**6.34**	**5.98**	**CUB-like domain, nematode specific**
***dod-22***	**9.90**	**61.4 ± 33.1**	**22.35**	**6.98**	**CUB-like domain/downstream of *daf-16*, nematode specific**
K08D8.4	9.45		5.84	1.11	CUB-like domain, nematode specific
***clec-68***	**9.42**		**13.57**	**8.24**	**C-type lectin**
***clec-67***	**9.06**		**13.12**	**8.12**	**C-type lectin**
***dod-21***	**8.91**		**15.28**	**14.07**	**CUB-like domain/downstream of *daf-16*, nematode specific**
***cdr-4***	**8.72**		**7.52**	**2.34**	**Glutathione S-transferase**
**C17H12.8**	**7.89**		**6.14**	**4.73**	**CUB-like domain, nematode specific**
*cdr-2*	7.68		7.00	1.53	Glutathione S-transferase
**Y41C4A.11**	**7.47**		**7.53**	**7.70**	**coatomer protein complex subunit beta**
**T24B8.5**	**7.34**		**4.03**	**4.13**	**ShK domain-like**
F35E12.8	7.18		7.27	2.01	CUB-like domain, nematode specific
C49G7.7	6.93		5.70	1.03	CUB-like domain, nematode specific
**K11H12.4**	**5.94**		**5.14**	**2.71**	**DUF274, nematode specific**
*Cyp-13A5*	5.93		2.09	1.01	cytochrome P450
C31A11.5	5.84		4.26	1.64	acyltransferase
*pme-5*	5.68		3.01	1.09	poly(ADP-ribose) polymerase (PARP)
*ugt-19*	5.43		8.95	0.71	UDP-glucuronosyl/UDP-glucosyltransferase
**T24C4.4**	**4.94**		**7.74**	**2.18**	**nematode specific**
Y51A2B.1	4.44		9.44	1.38	Predicted riboflavin biosynthesis protein
***gst-38***	**4.41**		**2.71**	**2.78**	**Glutathione S-transferase**
C17H12.6	4.07		4.34	2.42	CUB-like domain, nematode specific
***clec-4***	**4.05**		**16.41**	**4.15**	**C-type lectin/CUB domain**
C34H4.2	4.05		5.87	1.59	DUF274, nematode specific
Y94H6A.10	4.03		3.51	0.92	nematode specific
M02F4.7	4.01		4.77	1.53	C-type lectin
F53B2.5	3.85		2.81	1.33	Importin alpha-1 subunit
**C32H11.4**	**3.82**		**3.94**	**2.69**	**CUB-like domain, nematode specific**
F40F12.7	3.66		3.27	1.33	CREB binding protein/P300
T16G1.6	3.65		1.30	0.71	Predicted small molecule kinase/DUF227
T19D12.4	3.63		4.11	1.65	von Willebrand factor type A
***lys-2***	**3.52**	**3.8 ± 1.2**	**4.90**	**3.55**	**lysozyme**
**F49F1.7**	**3.46**		**5.24**	**4.64**	**ShK domain-like, Secreted surface protein**
***arf-1.1***	**3.29**		**4.01**	**2.11**	**ADP-ribosylation factor**
C10C5.2	3.24	3.7 ± 1.2	3.81	1.52	Cyclin-like F-box, nematode specific
F36G9.12	3.18		4.13	2.07	acyltransferase
***dod-17***	**3.15**		**4.55**	**2.95**	**CUB-like domain/downstream of *daf-16*, nematode specific**
**ZK896.5**	**3.15**		**3.79**	**2.68**	**CUB-like domain, nematode specific**
***dod-24***	**3.04**		**3.07**	**3.19**	**CUB-like domain/downstream of *daf-16*, nematode specific**
***egl-1***	**2.39**	**5.0 ± 0.3**	**2.91**	**2.49**	**BH3 domain-only protein, programmed cell death activator**

Genes were annotated according to Wormbase [31]. Bold type denotes genes significantly upregulated at least 2 fold 2 h and 6 h after IR. Values correspond to fold changes. Exemplary confirmation of transcriptional induction by qRT-PCR is shown.

**Table 2:** Checkpoint dependent and tissue specific induction of IR-induced genes.

	qRT-PCR					GeneChip	
		
**gene**	**N2 wild type**	***mrt-2(e2663)***	***cep-1 (lg12501)***	**N2 wild type**		**N2 wild type**	***cep-1 (lg12501)***
			
				germlines	embryos		
F49F1.6	39.34	45.38	49.03	ND	2.43	155.32	98.58
*dod-22*	12.06	6.29	10.54	ND	ND	9.90	8.55
*lys-2*	5.28	5.28	5.43	5.46	5.66	3.52	3.00
*pme-5*	2.00	4.88	1.37	1.03	1.78	5.68	6.82
Y47G7B.2	2.39	1.68	-1.01	1.23	2.27	1.78	1.01
*egl-1*	3.93	1.89	-1.61	6.96	-6.19	2.39	-1.20
*ced-13*	81.43	2.99	-1.30	-1.43	21.86	1.73	1.00

Quantitative RT-PCR was performed on samples taken 2 h after gamma-irradiation with 120 Gy. GeneChip refers to the Affymetrix GeneChip experiment using worms 2 h after X-ray treatment. Values correspond to fold changes.

**Figure 1:**
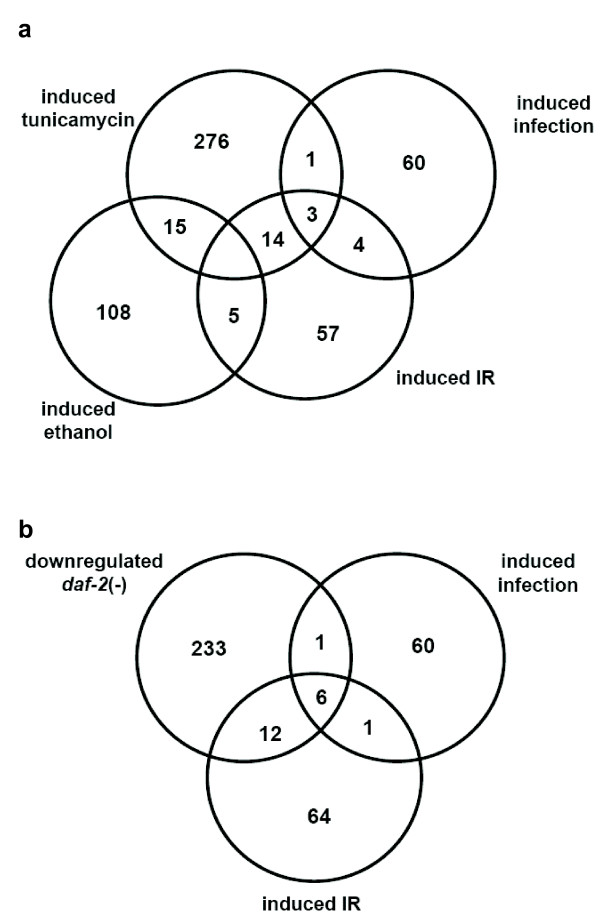
**Overlap of IR-induced genes with genes regulated by stress and aging**. (A) Venn diagram comparison of genes induced at least two fold in response to 120 Gy of X-ray treatment with genes induced in response to treatment with tunicamycin [19] and ethanol [33] as well as in response to bacterial infection [20](B) Comparison of IR-induced genes with genes regulated in a *daf-2 *dependent manner [21] and genes induced in response to bacterial infection [20].

We next wished to more closely analyze IR-induced genes and started by looking for overlaps between our dataset of induced genes with published *C. elegans *expression profiles (Figure 1, See Additional file 3). We found significant overlaps with the transcriptional response to various stress conditions and to the transcriptional response elicited by infections with bacterial worm pathogens. The biggest overlap with 17 out of 83 IR-induced genes occurs with genes induced by tunicamycin, an inhibitor of protein glycosylation which results in the activation of the unfolded protein response (Figure 1) [19]. Amongst the genes present in both datasets, two are annotated as glutathione S-transferases and two as C-type lectins. Furthermore, amongst the few genes known to be induced by ethanol treatment five are also induced upon IR. Interestingly three of these proteins share a domain of unknown function (DUF) 227, that occurs in four IR-induced genes and which is encoded in only 28 genes of the *C. elegans *genome. The next overlap (10 out of 84 IR-induced genes) is between genes induced by the *C. elegans *bacterial pathogen *Microbacterium nematophilum *that leads to the transcriptional induction of 68 genes, many of which have been shown to be necessary to ward off persistent bacterial infections [20]. Three of these genes encode a CUB-like domain, which represents a nematode specific domain of unknown function related to CUB domains (for complement C1r/C1s, Uegf, Bmp1 domains). CUB domains are structural motifs of approximately 110 residues found almost exclusively in extracellular and plasma membrane associated proteins, many of which are developmentally regulated (Figure 1). Since many IR upregulated genes appear to be antimicrobial or involved in stress response we also compared expression of the IR-induced genes with genes regulated by the insulin/IGF-1 signalling (IIS) pathway (Figure 1, See Additional file 3). Attenuation of IIS, for instance though *daf-2 *IGF receptor inactivation leads to extended longevity that is accompanied by increased stress resistance. *daf-2 *IGF receptor inactivation leads to life span extension mainly through activation of the *daf-16 *FOXO transcription factor leading to the activation of genes involved in cellular stress response and antimicrobial defence [21]. We found that of the 83 genes induced at least 2 fold upon IR, 18 are also *daf-2 *induced (downregulated in *daf-2(-) *and upregulated in *daf-16(-)*). Interestingly, knockdown of 4 of these genes, namely the CUB-like domain containing *dod-17*, *dod-21*, *dod-22 *and *dod-24*, resulted in an extension of life span [21]. The overlap in gene expression changes in response to DNA damage and longevity regulation suggests that both regimes induce a common stress response.

**Figure 2:**
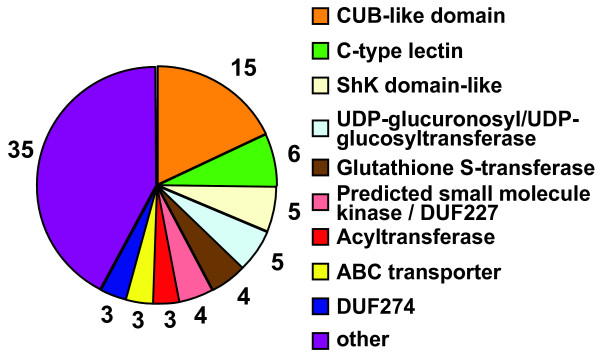
**Domains enriched in IR-induced genes**. Genes induced more than 2 fold 2 h after X-ray treatment were analyzed. The labels denote the number of proteins containing the respective domain.

Analyzing the domain structures of all IR-induced genes we found that genes encoding CUB-like domains are highly enriched upon IR, with 15 out of 60 such genes being induced more than 2 fold (Figure 2, See Additional file 4). Other domains enriched are C-type lectins (6/160), UDP glucuronosyl/UDP glucosyl transferases (5/of ~80), genes encoding Shk-like domains (5/116), glutathion S-transferases (4/66), ABC transporters (3/58), and genes encoding domains of unknown function (DUF) DUF227 (4/28) and DUF 274 (3/23) (Figure 2). Interestingly, many of the IR-induced genes are likely to be correlated with the detoxification of xenobiotics, a program which is often coordinated by transcriptional regulation [22]. Xenobiotics are generally metabolized and exported in three steps [22]. In phase I molecules are activated either through oxidation, reduction or hydrolysis. In phase II, activated metabolites are then conjugated to polar molecules to reduce their hydrophobicity, before they are finally exported as part of "phase III". Potential IR-induced phase I enzymes are the cyp-13A5 cytochrome P450, the Y51A2B.1 aldehyde dehydrogenase and possibly the C52A10.1 carboxylesterase. Examples of IR-induced phase II enzymes are 4 glutathione S-transferases and 5 UDP-glucuronosyltransferases and 2 acyltransferases, and three IR-induced ABC transporters represent "phase III" (Figure 2).

**Figure 3:**
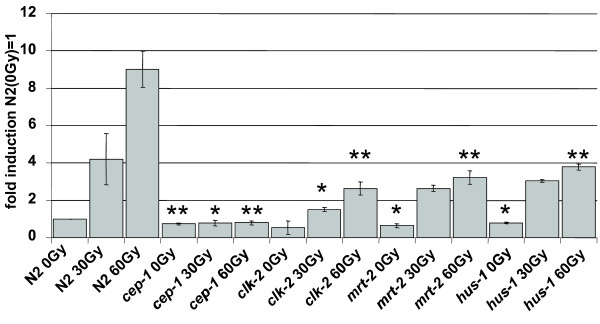
**Induction of *egl-1 *is fully dependent on *cep-1 *and partially dependent on DNA damage checkpoints**. Samples were taken 2 h post X-ray treatment and expression levels quantified by qRT-PCR. Expression levels were normalized to gamma tubulin (*tbg-1*) and compared to untreated wild type controls. Averages of three experiments are shown (error bars = SEM). Asterisks indicate statistically significant differences to the corresponding wild type samples (single asterisk p < 0.05; double asterisk p < 0.01).

To test whether IR dependent genes require the DNA damage checkpoint for their transcriptional induction, we tested the induction of selected genes in a *mrt-2 *checkpoint mutant. *mrt-2 *acts as part the 9-1-1 complex upstream of *cep-1*. In contrast to the *cep-1 *dependent induction of *egl-1 *and *ced-13*, all other IR induced genes tested were not dependent on *mrt-2 *thus further indicating that known DNA damage response pathways are not required for the transcriptional induction of most IR-induced genes (Table 2). Interestingly, we observed that the transcriptional induction of *egl-1 *and *ced-13 *is not entirely dependent on *mrt-2*, a result we confirmed by analyzing *hus-1 *and *clk-2 *checkpoint mutants (Figure 3). *hus-1 *encodes for a subunit of the 9-1-1 complex while the *C. elegans clk-2 *DNA damage checkpoint gene acts in a pathway parallel to the 9-1-1 complex [1,3]. Our results indicate that while *egl-1 *transcription is still induced by IR in checkpoint mutants, the level of *egl-1 *transcript tends to be reduced both before and after IR as compared to wild type (Figure 3). These results are consistent with a recent report by Quevedo et al. [23] and indicate that *egl-1 *transcription is dependent on functional DNA damage checkpoints even in the absence of IR, suggesting the presence of a low amount of constitutive DNA damage or a basal level of DNA damage signalling. This is in contrast to *egl-1 *transcription in *cep-1 *mutant worms where *egl-1 *transcription is comparable to wild type levels in the absence of IR treatment but *egl-1 *transcript levels are not induced upon IR [24]. These results suggest that DNA damage can also be sensed independently of *hus-1*, *mrt-2 *and *clk-2 *DNA damage checkpoint genes, or that the residual *egl-1 *induction may be due to redundancy between *hus-1/mrt-2 *and *clk-2*.

Given that *cep-1 *and *egl-1 *are known to effect DNA damage induced apoptosis, a response that only occurs in the *C. elegans *germ line, we wished to determine whether *egl-1*, *ced-13 *and other IR responsive genes are induced in the *C. elegans *germ line or in somatic tissues, which in adult worms are entirely postmitotic. To address this we compared transcriptional induction of select IR-induced genes between total worm lysates, isolated germ lines and isolated embryos (Table 2). Two out of three IR dependent but *cep-1 *independent genes tested were transcriptionally induced in whole worm lysates and in embryos but not in germ lines (Table 2). As expected we found that *egl-1 *is induced in germ lines and total worms. Surprisingly, we found *ced-13 *transcriptional induction only in total worm extracts and embryos (Table 2), but not in isolated germ lines that contained detectable *ced-13 *both with and without IR treatment (data not shown). We previously found that *ced-13 *is not induced in *glp-4 *worms that do not contain a germ line and embryos [11]. These results hint towards an unexpected role of *ced-13 *in DNA damage responses during embryogenesis. Furthermore, these data are consistent with the notion that *cep-1 *might transcriptionally activate target genes outside of germ line tissues, which is consistent with its reported expression during embryogenesis. As *ced-13 *transcriptional induction in embryos is *cep-1 *dependent we also asked whether this induction depends on *mrt-2*. *ced-13 *induction by IR was dramatically reduced in *mrt-2 *mutants indicating that both *cep-1 *and *mrt-2 *can act upstream of *ced-13 *in embryos (Table 2).

Given that *cep-1 *is the only *C. elegans *p53 family member and given that mammalian p53 family members p63 and p73 besides affecting apoptosis also have developmental functions we asked whether *cep-1 *might also have a global transcriptional role in worms independent of IR. A recent manuscript reported that hundreds of genes are induced by UV irradiation and dependent on *cep-1 *based on cDNA expression arrays that cover approximately 60% of the genome. In the same experiment 28 genes were reported to be *cep-1 *dependent irrespective of UV treatment [25]. To confirm these results and also to test for *cep-1 *dependent genes using sensitive genome wide Affymetrix GeneChip arrays we searched for *cep-1 *dependent genes using two multiply backcrossed *cep-1 *deletion strains, *cep-1(lg12501) *and *cep-1(gk138)*. The *lg12501 *allele takes out the C-terminus of CEP-1 [24] while the *gk138 *allele also takes out the CEP-1 DNA binding domain. We have also confirmed by western blot that no CEP-1 protein is present in either of the mutant strains (see Additional file 5). Testing for *cep-1 *dependent genes in the *cep-1(gk138) *allele we could only confirm 1 of the 28 *cep-1 *regulated candidates even though only one candidate gene was below detection levels on the Affymetrix arrays. When we considered only the genes that were differentially regulated independent of irradiation in our Affymetrix arrays, none of the 123 genes whose expression appeared to depend more than 1.5 fold on the *cep-1(gk138) *allele was reproducible in the *cep-1 (lg12501) *strain. Likewise, none of the 87 genes that appeared to be *cep-1(lg12501) *dependent were reproducible in the *cep-1(gk138) *strain. These results stress the importance of using at least two independently derived alleles when analyzing global transcriptional dependencies. Even when multiply backcrossed mutant worm lines are analyzed, potentially remaining background mutations can lead to observable transcriptional changes in highly sensitive Affymetrix microarrays. Of the IR-induced and *cep-1 *dependent genes only two, namely *egl-1 *and Y47G7B.2, whose expression profile we confirmed by qRT-PCR analysis (Table 2) was expressed in a *cep-1 *dependent manner in both *cep-1 *loss of function alleles. Albeit *ced-13 *was not considered X-ray induced as the signal was not significantly above background levels, we detected induction when looking at the absolute values on the array and confirmed this to be significant by qRT-PCR analysis and in the arrays probed with gamma-ray treated worms (Table 2). Y47G7B.2 was also induced more than 1.5 fold by IR in three independent microarray experiments using both *cep-1 *alleles and its expression profile was confirmed by qRT-PCR analysis. Y47G7B.2 encodes for an apparently *Caenorhabditis *specific gene, with several *C. elegans *and one *C. briggsae *paralog, while orthologs in the parasitic nematode *Brucia malai *could not be identified. We could not detect any obvious DNA damage induced apoptosis defect or any other developmental defect upon RNAi of this gene (data not shown). In summary, our data suggest that there are very few genes globally regulated by *cep-1*.

## Discussion

We show that multiple genes are induced by IR. Interestingly, the vast majority of these genes are neither related to DNA repair, DNA damage checkpoint signalling or apoptosis. We rather suggest that many of these genes tend to be expressed in somatic, non-proliferative worm tissues and might be related to general stress responses consistent with a common set of genes induced upon DNA damage and *daf-16 *activation. In line with this notion we did not find DNA damage induced germ cell phenotypes such as enhanced IR sensitivity, or defects in DNA damage sensing associated with RNAi inactivation of IR-induced but *cep-1 *independent genes (data not shown). Surprisingly, we found very few genes regulated by CEP-1. While CEP-1 is widely expressed in embryos and in the germ line, it is possible that some *cep-1 *dependent genes are only expressed in very few cells, making it impossible to detect subtle changes on expression arrays analyzing whole worm extracts, although about 50% of the cells in adult worms are germ cells. Nevertheless the expression profiles contrast with similar experiments performed on *daf-16*. DAF-16 is a forkhead transcription factor negatively regulated by insulin signalling and implicated in stress response and longevity regulation. Dozens if not hundreds of genes have been found to be transcriptionally regulated by *daf-16 *[21]. Besides a nematode specific gene we found that only *egl-1 *and *ced-13 *are upregulated by IR and transcriptionally dependent on *cep-1*. These data argue that DNA damage induced apoptosis of germ cells might be the evolutionary most ancestral DNA damage response function of the p53 family. There is an ongoing discussion as to which of the mammalian p53 family members is the most ancestral. Albeit p63 and p73 are implicated in developmental control most notably in epithelial development, stem cell maintenance and neurogenesis, they also impinge on DNA damage response regulation [10]. p63 and p73 cooperate in tumour suppression and their over-expression can affect induction of p53 response genes and apoptosis. The deletion of both genes in mouse embryonic fibroblasts can lead to defects in DNA damage induced apoptosis similar to p53 mutations [26]. Recent data indicate that an isoform specific knockout of p63 leads to defective DNA damage induced germ cell apoptosis in female mice [10]. This phenotype, reminiscent of *cep-1*, together with the p63 DNA binding domain being more closely related to *cep-1 *lead to the notion that p63 might be the closest mammalian CEP-1 homolog. This argument is also in line with recent structural studies on CEP-1 that reveal that CEP-1 contains a SAM domain which is retained in p63 and p73 but absent in p53 [14]. Furthermore, CEP-1 forms dimers and not tetramers like its vertebrate p53 family members and dimerization is facilitated by the SAM domain. These results suggest that *cep-1 *might be the primordial p53 member among nematodes, arthropods and vertebrates [14]. In this model, which assumes that the coelomata model of evolution is correct, dimerization would be primordial with tetramerization having evolved independently in arthropods and vertebrates where three p53 members evolved. Interestingly, the basic non-bilaterian sea anemone *Nematostella vectensis *contains three p53 like molecules, one of which was implicated in UV induced apoptosis [27]. It is clear that these three p53s evolved independent of the vertebrate p53, p63 and p73 although it is surprising that *Nematostella *p53 DNA binding domains are more closely related to vertebrates as compared to worm and fly p53 like proteins, a feature shared with many other proteins [28].

## Conclusion

Our data indicate that the apoptotic response to DNA damage is regulated through CEP-1 mediated transcriptional induction of *egl-1 *and *ced-13*, whereas other DNA damage responses such as cell cycle arrest and DNA repair, which are also found in unicellular organisms such as yeast, might instead be mediated through posttranscriptional modifications of checkpoint proteins. Furthermore, we reveal that most of the transcriptional response to IR, which is independent of CEP-1, appears to occur in the post mitotic soma of the worm and is part of a general stress response that partially overlaps with the unfolded protein response and DAF-16 mediated longevity assurance. Our transcriptional data and the absence of an overt developmental phenotype associated with *cep-1 *deletions [6,7] suggest that DNA damage induced apoptosis might be a primordial function of the p53 family. Later in evolution other p53 functions, such as the transcriptional upregulation of DNA repair proteins or the inclusion of cell cycle control by p21, which has a developmental role in *C. elegans *[29], might have evolved. The triplication of p53 in vertebrates might then have allowed p63 and p73 to acquire developmental functions, while retaining a role in DNA damage induced apoptosis.

## Methods

### *C. elegans *strains & maintenance

Worms were maintained at 20°C on NGM agar plates according to standard protocols [30]. Alleles used were: LG I: *cep-1(lg12501), cep-1(gk138), hus-1(op244)*; LG III: *mrt-2(e2663), clk-2(mn159)*. For sequence information see Wormbase [31].

### Microarrays

Approximately 1000 age synchronized young adult hermaphrodites (24 h post the L4 larval stage) were irradiated with 0 Gy or 120 Gy of X-rays using a Stabilipan (Siemens) setup and RNA extracted 2 h post treatment (for *cep-1(lg12501) *and wild type). For the gamma-ray experiments, young adults were irradiated with 0 Gy or 120 Gy using an IBL 437C (CIS bio international) and RNA extracted 2 h post treatment (*cep-1(lg12501) *and wild type worms) or 6 h post treatment (*cep-1(gk138) *and wild type). For each condition and strain 3 samples were analyzed, except for samples taken 2 h after gamma-irradiation where only 2 samples were analyzed. RNA was extracted using TRIZOL (Invitrogen) according to the manufacturer's protocol. For the gamma-ray experiment, the RNA was purified from TRIZOL using the RNeasy Mini Kit (Qiagen) as described for qRT-PCR (see below). Approximately 20 μg of total RNA was obtained from each sample. RNA was not amplified prior to labelling. Synthesis of double stranded cDNA and biotin labelled cRNA was performed according to the instructions of the manufacturer (Affymetrix, USA). Fragmented cRNA preparations were hybridized to *C. elegans *genome oligonucleotide arrays (Affymetrix), using Affymetrix hybridization Oven 640 (Affymetrix, USA), washed, and subsequently scanned on a GeneChip Scanner 3000 (Affymetrix, USA). Initial data extraction and normalization within each array was performed by means of the GCOS software (Affymetrix). Data intensities were log transformed and normalized within and between arrays with the quantile normalization method, and two-tail, pair wise analysis or a two-way analysis of variance was employed by means of the Spotfire Decision Site software package 7.2 v10.0 (Spotfire Inc., MA, USA) to extract the statistically significant data from each group of worms indicated in this study. A gene was considered for analysis if the hybridization signal for the corresponding probe was deemed to be present on the array (significantly above background levels (p < 0.05)) in at least 2 of 3 relevant arrays in the X-ray experiment. A gene was considered induced/repressed if the difference in expression levels was significant (p < 0.05) compared to the control. To be considered the change in expression had to be at least 2 fold compared to the control, except where indicated. Reproducibility of the array experiments was further confirmed with Pearson correlation (See Additional file 6). Genes were annotated using Wormbase [31].

### Quantitative real time RT-PCR

RNA was extracted from 25 worms for each condition and strain analyzed, using TRIZOL and Purelink Micro-to-Midi columns (Invitrogen) according to the manufacturer's specifications. To facilitate extraction, worms were disrupted in TRIZOL with 0.7 mm zirconia/silica beads (Biospec Products) using a Mini-Beadbeater 8 (Biospec Products) at maximum speed for 30 seconds. To measure expression in the germ line, worms were dissected in PBS and RNA was extracted from 6 isolated germ lines for each condition assayed. To measure expression in embryos, ca. 20 gravid adult worms were dissected in PBS and the embryos transferred to TRIZOL. Extraction was performed using the same protocol as described for whole worms. RNA concentrations were measured and equal amounts reverse transcribed using the Quantitect kit (Qiagen). Between 0.2 and 0.5 μl of the reverse transcription reactions were used for quantitative real time PCR using the MesaGreen mix (Eurogentec) on an iCycler iQ5 (Biorad). Cycling conditions were: 1× [5 min 95°C] and 50× [15 s 95°C, 20 s 60°C, 40 s 72°C] fluorescence was measured after each 72°C step. Relative expression levels were determined according to Pfaffl [32], using *tbg-1 *transcript as a standard. Experiments were performed in triplicate.

The following primers were used:

F49F1.6: 1730 5'-CTTGTGGAATATGCCATCAG-3' and 1731 5'-GGGCATTGTATCTTAACAGC-3'; *dod-22*: 1732 5'-GGCTACCATTTCCAAACATAG-3' and 1733 5'-CTCCTTCAAATACAAGAGCAC-3'; lys-2: 1736 5'-TCTGGATTCAGGTTACTTCC-3' and 1737 5'-CCAAGAACATTCCAATACCA3'; Y47G7B.2: 1746 5'-ATTTCTCGTATACGATGGTTGC-3' and 1747 5'-TTCGGACAATTTCTGAATCTCC-3'; *tbg-1*: 1760 5'-AAGATCTATTGTTCTACCAGGC-3' and 1761 5'-CTTGAACTTCTTGTCCTTGAC-3'; *egl-1 *1762 5'-CCTCAACCTCTTCGGATCTT-3' and 1763 5'-TGCTGATCTCAGAGTCATCAA-3'; *ced-13*: 1764 5'-GCTCCCTGTTTATCACTTCTC-3' and 1765 CTGGCATACGTCTTGAATCC-3'; *pme-5*: 1893 5'-ATTACTGATCCATCGCTCTTCTC-3' and 1894 5'-CCAACTCAATCGGATTCGGA-3'.

One primer in each pair was designed to bind at the site of an exon-exon boundary to ensure that only cDNA corresponding to processed mRNA was amplified. Each primer pair was tested with a logarithmic dilution of a cDNA mix to check for efficient amplification.

### Western Blot

To make protein extract, worms were boiled for 20 min in LDS sample loading buffer (Invitrogen). The worms used were a mixed population consisting of all larval stages and adults. Protein extracts were run on 4–12% Bis-Tris NuPAGE gels (Invitrogen) and then blotted to a PVDF membrane (Millipore). Gel electrophoresis and blotting were done using an XCell tank (Invitrogen) according to the manufacturer's protocols. After transfer the membrane was blocked for 1 h at room temperature in blocking buffer (PBS + 0.1% Tween20 supplemented with 5% milk powder). Binding of the primary antibodies was performed over night at 4°C. Incubation with secondary antibodies was performed for 1 h at room temperature. All washing and binding steps were done in blocking buffer. The blot was probed with anti CEP-1 antibody raised in goat [24]. Alpha-tubulin was used as a loading control and probed with anti alpha-tubulin monoclonal antibody (clone DM 1A from Sigma). Secondary antibodies used were horseradish peroxidase coupled donkey anti-goat and goat anti-mouse (both Jackson ImmunoResearch). Immobilon Western Chemiluminescent HRP Substrate (Millipore) was used for detection.

## Authors' contributions

SG and BS did all experiments and the bioinformatics analysis and helped with writing this manuscript. KG and JR supplied the first arrays, set up an array facility and helped with setting up the initial experiments. AG raised funds, supervised the project and wrote the manuscript.

## Availability and requirements

The original microarray data is available at  .

## Supplementary Material

Additional file 1 **Expression levels for all Affymetrix probes in all experiments**. Absolute expression levels are derived from normalized log values. Presence denotes the number of arrays in each experiment with a hybridization signal significantly above background levels (p < 0.05). Click here for file

Additional file 2 **Expression levels for Affymetrix probes upregulated at least 1.5 fold 2 hours after X-ray treatment**. Absolute expression levels are derived from normalized log values. Presence denotes the number of arrays in each experiment with a hybridization signal significantly above background levels (p < 0.05). Click here for file

Additional file 3 **Names of the genes in **Figure 1. Overlap of IR-induced genes with genes dependent on *daf-2*, as well as genes induced in response to stress factors other than IR. Click here for file

Additional file 4 Names of genes in Figure 2. Click here for file

Additional file 5 CEP-1 western blot of wild type and *cep-1 *deletion worms. Click here for file

Additional file 6 Pearson correlation of the individual arrays of the biological replicates used. Click here for file
