# Supplementary figures and images for: Transcriptional profiling in C. elegans suggests DNA damage dependent apoptosis as an ancient function of the p53 family
Source: BMC Genomics. 2008 Jul 15;9:334. doi: 10.1186/1471-2164-9-334 (PMC2491638; doi:10.1186/1471-2164-9-334)

*wild type*

*cep-1(gk138)*

*cep-1(lg12501)*

CEP-1

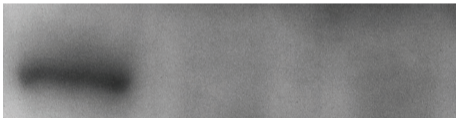

alpha-tubulin

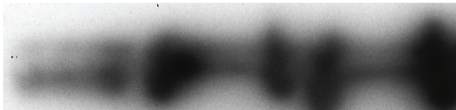

Supplement: Additional file 5 — CEP-1 western blot of wild type and cep-1 deletion worms. [file 1471-2164-9-334-S5.pdf]
